# Recent Advances in Vehicle Driver Health Monitoring Systems

**DOI:** 10.3390/s25061812

**Published:** 2025-03-14

**Authors:** Lauris Melders, Ruslans Smigins, Aivars Birkavs

**Affiliations:** Faculty of Engineering and Information Technologies, Latvia University of Life Sciences and Technologies, LV3001 Jelgava, Latvia; ruslans.smigins@lbtu.lv (R.S.); aivars.birkavs@lbtu.lv (A.B.)

**Keywords:** advanced wearable devices, remote health devices, driver health monitoring, biosensors

## Abstract

The need for creative solutions in the real-time monitoring of health is rapidly increasing, especially in light of health incidents in relation to drivers of motor vehicles. A sensor-based health monitoring system provides an integrated mechanism for diagnosing and managing in real time, enabling the detection, prediction, and recommendation of treatment and the prevention of disease onset. The real-time monitoring of driver’s health represents a significant advancement in the assurance of driver safety and well-being. From fitness trackers to advanced biosensors, these devices have not only made healthcare more accessible but have also transformed how people interact with their health data. The purpose of this scoping review is to systematically collect and evaluate information from publications on driver health monitoring systems to provide a comprehensive overview of the current state of research on wearable or remote sensor technologies for driver health monitoring. It aims to identify knowledge gaps that need to be addressed and suggest future research directions that will help to fill these gaps. This approach involves the topic of vehicle safety and healthcare and will contribute to the advancement of this field. By focusing on the real-time monitoring of health parameters in an automotive context, this review highlights the potential of different types of technologies to bridge the gap between health monitoring and driver safety.

## 1. Introduction

Driver health detection techniques have the potential to significantly improve road safety measures and reduce the number of accidents. These approaches gather information about the health of the driver from the vehicle using a variety of sensor devices; this is effective as driving is the most widely used means of transport in the world. This high usage demand is the motivation for the automotive industry to improve the life cycle of its products. Vehicle design, assembly operations, and vehicle technology have all been improved in recent decades. In recent years, the areas that have undergone the most immediate change and challenge have been customer service and environmental impact.

Interest in technology and telemedicine devices, as a biosensor, has increased significantly and brought these changes in the healthcare environment, particularly with the combination of technologies that have resulted in the creation of compact devices and improvements in data transmission speeds and affordability. A wide range of digital biomedical devices are currently available for the assessment of various indices of human activity and ability. The number of connected devices in the world is expected to increase from 300 million to more than 1 billion by the year 2024 [1].

Despite all the attractive capacities that biosensor devices possess, which have facilitated the extensive integration of biological and life science disciplines, there are several applications that have emerged. Key challenges include the advancement of personalized medicine, the facilitation of rapid diagnostics, the development of real-time monitoring applications, and the enhancement of preventive healthcare. However, especially with an aging population, physiological problems pose a significant risk to vehicle driver safety. As the level of automation in vehicles increases, so does the need for robust driver monitoring systems, as there must be a guarantee that the driver can intervene at any time. The quality of health for drivers, particularly those operating buses and heavy trucks, represents a significant contributing factor to road accidents as these drivers frequently encounter monotonous driving conditions. As a machine, the vehicle must offer the user comfort and safety, but despite all the technological improvements made by researchers and the industry, the vehicle cannot prevent accidents caused by driver error. In essence, monitoring heart rate patterns can offer valuable insights in terms of the driver’s physiological condition and level of alertness, as well as identifying the presence of any health symptoms. In order to operate a vehicle in a safe and appropriate manner and to respond effectively to potential road hazards, it is essential that drivers maintain their full attention and optimal health. To provide continuous health monitoring and address the growing demand for health services, it is imperative that accessible, cost-effective, and user-friendly healthcare solutions are implemented [2], and monitoring driver state helps to protect traffic safety and the transportation industry overall.

Advanced technologies, including autonomous vehicles [3,4,5], advanced driver assistance systems (ADASs) [6,7], and Vehicle-to-Everything (V2X) [8,9], are transforming the automotive landscape. The innovations offered are benchmarks for road safety, driver well-being, and the automotive industry overall. Features like lane departure warning, blind spot detection, and automatic emergency braking reduce accidents, enhancing driver safety and confidence. This shift is driven by advanced technologies, making driving safer and more efficient. With that said, recent studies have introduced innovative approaches to further highlight not only biosensor usage in different scenarios but also road safety, focusing on technology integration, and driver behavior analysis. Advancements in AI-driven driver assistance systems show great premise in improving road safety. By detecting hazardous behaviors, such as distracted driving, reckless lane changes, or sudden braking, these mentioned systems can help to prevent accidents, saving lives and reducing damage costs. The integration of sensors and machine learning (ML) algorithms enables real-time monitoring and alerts vehicle drivers. This allows drivers to adjust their behavior and lower the risk of accidents, ensuring a safer driving environment in the traffic safety context [10]. In modern vehicles, all pedal positions and driving data are transmitted to the Controller Area Network (CAN) and can be assigned to the associated sources using reverse engineering methods. What drivers see or recognize can be recorded from the driver’s perspective using (eye-tracking) videos or photos.

Several studies also have been focusing on driver behavior monitoring and the analysis of different driving styles. Recently, another approach was used [11], where the authors assessed traffic hazard perception and the responsiveness of drivers. Distracted driving, defined as the use of electronic devices, especially mobile phones, while operating a vehicle, has been identified as a major contributing factor to vehicular collisions. With that said, a team of Swedish and Austrian researchers performed a systematical review on distracted driving behavior using smartphones [12].

Health monitoring systems in vehicles improve driving safety by tracking vital signs, including heart rate (HR) and respiration rate (RR). They can detect conditions like sleep apnea, heart disease, or stress. These systems alert drivers to potential concerns and can communicate with the emergency services during medical events. They also improve driver behavior and health by offering real-time health feedback. Tracking health data over time provides insights into the long-term well-being of drivers, especially those in the commercial sector or with chronic conditions.

ADASs are a growing market trend, driven by the increasing demand for enhanced road safety features. Technologies such as driver health and fatigue monitoring, which employ cameras and sensors to detect drowsiness and declining health, are becoming increasingly popular. These systems can potentially prevent accidents by alerting drivers to potential risks or even taking control of vehicles in critical situations. The integration of the ADAS into vehicular disputes is expected to improve road safety, reduce accidents, and enhance overall driving experiences [13].

**Figure 1:**
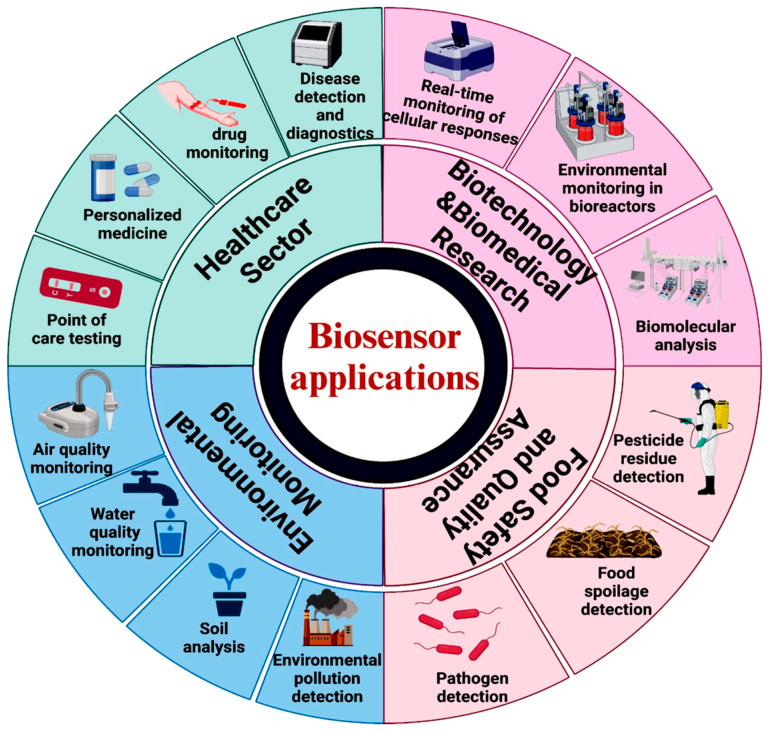
Various uses of biosensors in healthcare. Copyright MDPI (2024) [2].

On the other hand, technologies that facilitate digital tools are increasingly employed in the healthcare sector to enhance comprehensive human health monitoring. It can be argued that wearable and mobile-based sensors represent a crucial component of the emerging concept of biosensors (see Figure 1).

Therefore, this article will review the recent advances in driver health monitoring systems, using wearable and remote biosensors for health monitoring. In addition, the unsolved challenges for future researchers who specialize in a variety of areas (e.g., wearable sensors, device software, data analysis) will be under discussion briefly.

## 2. Methods

The paper is a review of the literature that was conducted regarding sensor technologies from the perspective of traffic safety; the purpose of which was to ascertain the feasibility of monitoring vehicle driver’s health using wearable devices and providing remote monitoring.

This review article aims to gather information on advancements in vehicle driver health monitoring solutions and to assess the present state of such devices more accurately, with a view to anticipating their future evolution. The focus will be on driver critical health indices measured by wearable and remote sensor vital signs sensing technologies, as well as their operating principles and specifications. In addition, the unresolved issues for future researchers specializing in various fields (e.g., biosensors, healthcare, transportation, etc.) are briefly discussed, also paying attention to future development forecasts.

*Inclusion criteria:* This review covers research works related to (1) technologies for driver health monitoring, (2) health indices for monitoring, and (3) commercial and noncommercial wearable remote devices for health monitoring.

*Exclusion criteria*: We discarded (1) sources not written in English, (2) non-peer-reviewed sources, (3) letters and reports, (4) conference and symposium proceedings, (5) and non-primary studies.

**Information Sources.** The keywords contained within the research questions were grouped into two categories or knowledge areas—traffic safety and healthcare—to ascertain the specific databases to be included in the review. In terms of the health sector, criteria for including research publications were put in place on the main health indices: SpO_2_ (blood oxygen saturation), blood pressure (BP), and heart rate (HR).

**Figure 2:**
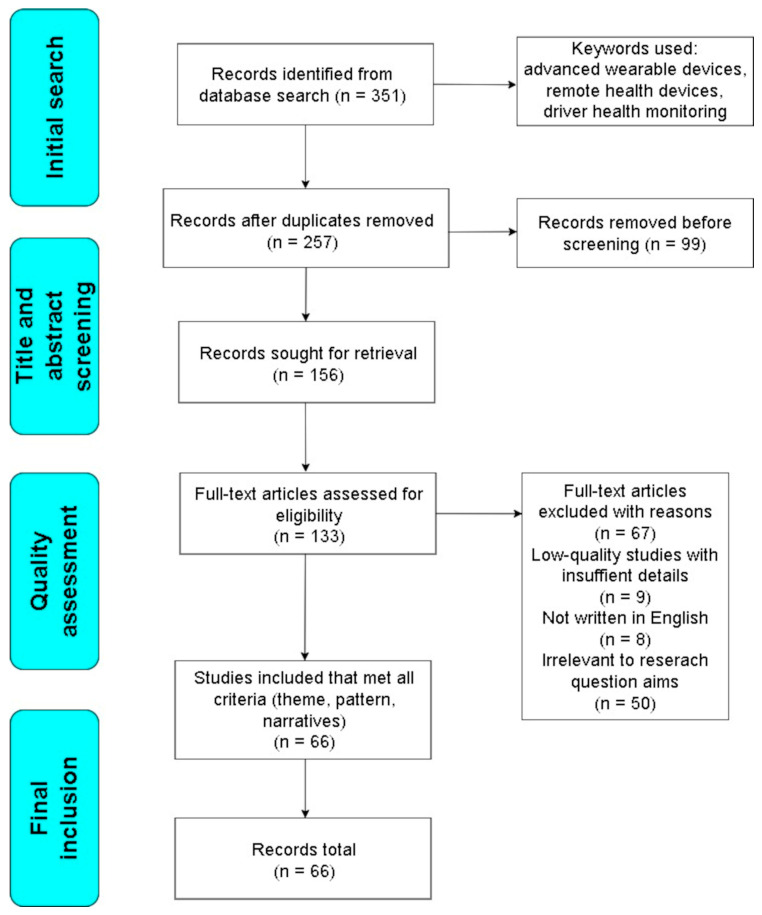
PRISMA flow diagram of the search strategy.

**Search strategy.** The search strategy used in this review was to combine keywords with Boolean operators (“and”, “or”) to limit the search results. To enhance reproducibility in the screening process, we ensured that each step was transparent, consistent, and systematically followed. Relevant studies were identified by searching through the PubMed^®^, Web of Science^®^, Institute of Electrical and Electronics Engineers^®^ (IEEE), and Science Direct^®^ databases. To identify other eligible studies, the reference lists of the original research articles and reviews on the topic were manually reviewed. We began compiling papers for this review in October 2024. As we were preparing this review, we compiled a second round of papers in February 2025 to incorporate the latest works published in 2025. With that said, Figure 2 indicates key article search actions, their inclusion/exclusion criteria, and details. After a thorough review of the academic papers, additional sources were excluded. These sources included reports from government agencies, reputable newspaper articles, and websites.

We used an AND rule to obtain combinations of the above meta-keywords (A) and (B). For each combination, we obtained the top 50 search results ranked by relevance. We did not consider any patent or citation-only search results (no content available online).

The combinations of search terms driver monitoring AND (SpO_2_ OR blood pressure OR heart rate) AND (Detection OR Estimation OR monitoring) AND (Physiological OR EEG OR EOG OR ECG OR face OR eyes OR behavioral) AND (real-time OR online) were used.

This study identified **351** articles after searching using the search keywords. Of this amount, **99** articles were filtered out by the duplication criteria using the Mendeley software application (Version 2.131.0). After the duplication procedure, 94 papers were removed since their research scope did not adhere to the criteria of the aim of our review. At the quality assessment stage, 67 records were removed, so the review finally comprised 66 records after applying the inclusion and exclusion criteria for eligibility.

However, we admit that the review process conducted in this work has some limitations. Due to the overwhelming number of papers published in this field in recent years, it is almost impossible to include all the published papers in the field of deep learning-based wearable human activity recognition in a single review paper. The selection of the representative works to present in this paper is unavoidably subject to the risk of bias. In addition, we may have missed the very first paper initiating or adopting a certain method.

To enhance the scientific contribution of this manuscript, each section includes an analysis of the reviewed articles, along with summary tables highlighting their key features. The structure of this review is as follows: Section 1 introduces the topics covered and explains the methodology used to select the scientific articles. Section 2 explains the specific objectives of the review, including the publication criteria used to select the scientific articles as sources of information. Then, Section 3 introduces the biosensor usage in vehicles, overall working principles on various sensors, and their recent advancements. Furthermore, use in the health monitoring context and legal framework are explained. In Section 4, common types and key parameters for health data acquisition methods are used to exploit wearable sensors and their optimization techniques for monitoring vehicle driver’s health. Considering usage in different variations—wristbands and smartwatches—ring-type sensors are summarized in table formats. Then, Section 5 explores the application of remote sensors in smartphones, as well as practical cases in patient monitoring, and refers to next-generation remote devices. Section 6 discusses the current limitations for future applications and provides a comparative analysis between wearable and remote sensors. Finally, Section 7 concludes this review, referring to future outlooks and perspectives in the biosensor field.

## 3. Fundamentals of Biosensors: Use in Vehicles, Working Principles, and Recent Advancements

### 3.1. Wearable Technologies for Driver Monitoring

The primary causes of road traffic accidents are driver error, characterized by excessive speed, misdirection of the vehicle, and other aberrant actions. To facilitate the transition to eco-driving, a novel mode that promotes considerate driving in all situations and concomitantly reduces mechanical risk and environmental impact from excessively polluting emissions is imperative. As outlined in the literature, there has been a significant volume of scientific contributions in the area of technology development, particularly looking at patient health analysis and monitoring technologies. With that said, the researchers in [14,15] discussed the advances in integrated wearable and implantable optoelectronic devices for healthcare. Advancements in technology have led to significant developments in the field of health monitoring, with particular emphasis on the use of wearable and implantable devices that are based on flexible and stretchable electronics. Also, the authors in [16] reviewed the function of wearable devices and summarized recent advances in the field of multifunctional wearable sensors, including individual sensors with different functions.

The popular belief that wearable devices have only developed relatively recently is erroneous. It is important to note that the concept of “smartness” has not always been synonymous with the processing of data on a chip. Rather, it has historically been defined by the delivery of superior experience for actual users. It is vital to assess a driver’s health state to ensure road safety and develop advanced monitoring systems, as there is a constant and growing increase in road accidents, even minor ones. The portable health monitoring market holds great promise around the world. However, the benefits of wearable devices will not be fully realized if there is a lack of uptake. Understanding the key drivers of wearable health technology adoption remains uncertain due to the paucity of studies on wearable health technology adoption [17].

Dangerous driving behaviors such as drowsiness or talking on the phone may seem commonplace in everyday life. However, these behaviors are the circumstances of many road accidents and pose a serious threat to road safety. It is therefore necessary to effectively detect dangerous driving behavior through scientific and technological means.

Recently, many researchers have studied the recognition of driving style and behavior itself, including aggressive, fatigued, or drowsy driving. A range of levels can be adopted for the study of driving behavior, including tactical, strategic, situational awareness, unmotivated behavior, and operational habits. Authors have used self-powered triboelectric sensors and a smart neck ring sensor system to monitor driver status. By collecting detailed information about the driver, the system can detect dangerous driving behavior, monitor the health status of the driver, and provide appropriate reminders to improve driving safety [18]. Another approach is where scientists from Morocco developed a technique for detecting a driver’s condition and driving style. This experimental research was conducted through the use of a monitoring unit that can be installed in any vehicle equipped with CAN (Control Area Network) technology, with the purpose of monitoring current driving actions. To define normal or aggressive driving, the authors combined the measurement of driving parameters such as engine speed, vehicle speed, accelerator pedal position, steering wheel angle, engine noise level, and fuel consumption [19].

To monitor driver activity behind the steering wheel, authors have proposed a simple yet effective framework for Wi-Fi-based driver activity monitoring. Activities such as eating, using a mobile phone or infotainment system, and gear stick use were executed. The benefit on this study is that it involves cost-effective solutions that do not need a sophisticated camera support to capture images or special hardware to be carried by users [20].

To illustrate driver drowsiness and detection in dynamic environments, sensor technology must be non-intrusive to avoid road vibrations. The authors of [21] proposed an ML approach for driver drowsiness detection using their physiological data. The findings indicate that the non-intrusive setting delivers accuracy that is comparable to that of a medical-grade device, with high accuracies (>92%). This new approach creates new ways for humans and machines to interact in vehicles, especially when it comes to monitoring drivers’ drowsiness in automated driving situations.

Just as important is driver fatigue detection, which is roughly divided into subjective detection and objective detection methods. Objective detection methods are often susceptible to external factors, such as road conditions and lighting levels, which can compromise accuracy. In contrast, subjective methods rely on physiological signals and have proven to be effective in accurately assessing driver fatigue and overall mental state. Electroencephalography (EEG) signals offer unique advantages due to their high temporal resolution and wearable and portable acquisition equipment, making them a leading solution for identifying fatigue-related driving conditions [22].

### 3.2. Working Principles

Wearable sensors provide the hardware component that captures multiple signals, including those of an activity, both physiological and environmental in nature. Advances in biometric technology have created sensors that track physical activity, offering valuable insights into health status. Human gait analysis is a prominent feature capable of detecting diseases like Parkinson’s and Alzheimer’s. Regularly checking your activity can help detect and treat health problems early and can detect vital signals from the human body, including blood pressure, heart rate, body temperature, throat moisture, or other relevant human body information such as joint activity and micro-expressions. Wearable devices do not require the driver to take any action, such as putting on the device, but instead assess physiological characteristics such as EEG, Electrooculogram (EOG), and Electrocardiogram (ECG) readings and detect fatigue by extracting facial features and data from webcams [23,24]. This section summarizes key sensing mechanisms being used in wearable biosensors.

A coil, also known as an inductor, is a passive electrical component that stores energy in a magnetic field when an electric current flows through it. It is made up of a conductor, like a copper wire, wound into a series of loops or turns. Meanwhile, a permanent magnet is a material that retains its magnetic properties and generates a persistent magnetic field without the need for an external power source. Coil wires are usually made of copper due to their high conductivity; however, permanent magnets contain iron, nickel, and cobalt materials. Coils are key to converting electrical energy into magnetic energy. They are essential in making electromagnets, inductors, and transformers. Meanwhile, permanent magnets provide a constant magnetic field and are used in electric motors and everyday magnetic devices. Overall, a coil and permanent magnet attract or repel each other, generating voltage. These sensors can be used in large quantities for multi-location sensing in the healthcare industry [25].

**Figure 3:**
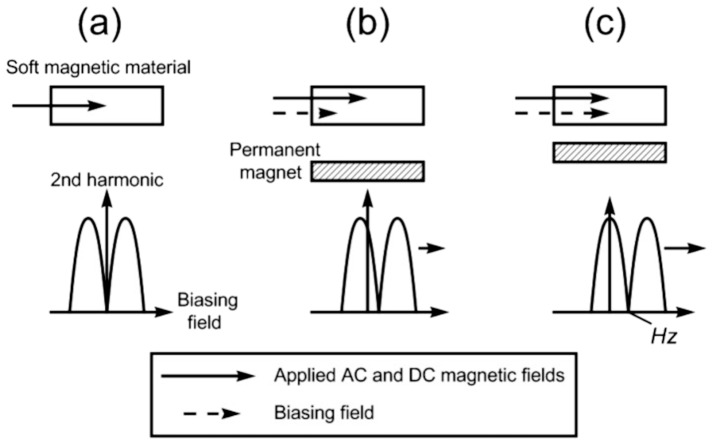
Operational principle of the magnetic field sensor. The harmonic field possesses a symmetrical nature and is dependent upon the presence of an applied direct current (DC) field (**a**). The positioning of a permanent magnet generates a biasing field, which results in a shift in the harmonic field pattern (**b**). As the separation distance between two magnetic elements decreases, the secondary biasing field experienced by a soft magnetic material increases accordingly. (**c**) Copyright MDPI (2008) [26].

Soft magnetic material, called the sensing element, was found to generate secondary magnetic fields. The specimen was exposed to a low-frequency alternating current (AC) magnetic field. Afterwards, secondary magnetic fields include higher-order harmonic modes (see Figure 3).

Ultrasonic sensors use sound waves to measure distance or detect objects. The functionality of these devices is based on the analysis of sound waves, measuring the time it takes for the waves to reflect off an object and return to the sensor. They are capable of detecting changes in physical and chemical properties through the use of body or surface acoustic waves [27]. Typically, they consist of a transmitter, receiver, control circuit, and transducer. In most cases, they are used for distance measurement, object detection, level sensing, or even in industrial production automation. Their advantages include non-contact measurements (i.e., no physical interaction is required), versatility (working in a variety of environments, including darkness, dust, or fog), and their relatively low cost and ease of integration. Nonetheless, it is important to note that environmental factors, such as temperature, have the capacity to influence the accuracy of the process. In comparison with magnetic sensors, which are typically constrained in their ability to record signals on the skin, ultrasound sensors possess the capacity to offer enhanced functionality. The material was meticulously divided into several centimeters, and a three-dimensional aperture was subsequently crafted to accommodate for the electronic components [28].

Electrochemical sensors represent a promising technique for the continuous monitoring of biological changes in sweat. An analysis of human sweat can provide valuable insights into various physiological processes and potential health concerns. They are electronic devices capable of detecting and measuring the presence of chemical substances through the conversion of a chemical reaction into an electrical signal. These sensors find extensive applications in fields such as gas detection, environmental monitoring, and medical diagnostics. Electrochemical sensors comprise a working electrode, where the target chemical reaction occurs; a counter electrode, which completes the electrical circuit; a reference electrode, which maintains a stable potential for accurate measurement; an electrolyte, which facilitates an ion exchange between the electrodes; and a gas-permeable membrane, which allows the target gas to enter while blocking interfering substances. Electrochemical sensors are key to detecting low concentrations of target gases. They are highly selective, able to distinguish target gases from interfering substances. Also, the lifetime of these sensors is typically 1–3 years, depending on usage and environmental conditions. They operate on reduction–oxidation reactions; in a medical context, they are widely used for glucose and sweat monitoring. The composition of sweat varies, but it typically contains four primary components: choline, lactate, glucose, and ascorbic acid.

Meanwhile, electrochemical biosensors are devices that utilize the electrical properties of these components to detect changes in sweat. These biosensors generally consist of three electrodes: a reference electrode (anode), a counter electrode (cathode), and a working electrode. Afterwards, electrochemical conversion elements can detect changes in an electrical current caused by ions or electrons being produced and used to measure the health status of the individual. A bioreceptor is an organic body that can detect a specific molecule with high sensitivity while remaining unaffected by other interferences. Examples of biorecognition components include enzymes, nucleic acids, antibodies, and microbes. A transducer is a device capable of converting the physicochemical properties (e.g., electrical current, voltage) of an electroactive entity into a measurable analytical signal. An amplifier detects and amplifies electrical signals, which are then sent to a signal processor. The signal is converted and prepared for display. The display unit reproduces curves, graphs, voltammograms, or numbers [29].

Temperature sensors are devices that combine biological components with temperature-sensing elements to measure temperature changes in biological systems. They are used in medical diagnostics, environmental monitoring, and biotechnology and consist of a biological element, a transducer, a temperature-sensing element, and a signal processor. When the biological element (cell, for instance) interacts with the target analyte (glucose, protein, pathogens), the temperature change is proportional to the concentration of the analyte. Then, the temperature-sensing element can detect the change in temperature caused by this reaction. In the field of medical diagnostics, they are employed for the monitoring of body temperature and the detection of diseases, such as fever and infection. One of the biggest limitations of this sensor is calibration for accurate measurements. With that said, temperature remains an essential physiological signal for human health, providing a direct diagnostic approach. Variations in body temperature are a consequence of metabolic alterations and can be incorporated into flexible circuits for wearable body temperature monitoring applications [30]. Furthermore, continuous body temperature measurements show premise and can provide vital information for predicting human diseases, for instance, in respiration [31] and even for trauma patient [32] monitoring.

Pressure and mechanical sensors are ideal for applications requiring high precision, such as medical fields. These sensors have been used for measurements such as heart rate, blood pressure, and RR. In most cases, they contain sensing elements which detect physical force (e.g., pressure, weight, or mechanical stress). Afterwards, a transducer converts this physical force to a measurable signal, mostly in electrical or optical format. After that, the signal processor analyzes the signal and displays or transmits the measured data to visual format. The sensing element is designed to respond to the applied force, with the specific type of sensor determining the way it does so. For instance, pressure sensors involve the deformation of a diaphragm or membrane under pressure, while strain gauges operate through the stretching or compression of a conductive material, thereby altering its resistance. Additionally, piezoelectric sensors are based on the generation of an electrical charge by a piezoelectric material when subjected to mechanical stress.

With that said, pressure sensors are divided into two types: stretch and pressure/tactile. Pressure sensors can detect motion by measuring mechanical deformation, which changes the electrical signal (piezoelectric or piezoresistive) [33]. Meanwhile, wearable stretch sensors are widely used to detect motion. They can be used in large motions (e.g., arm, hand, and knee motion) or small motions (e.g., chest motion during breathing) [34]. To be further developed, pressure sensing technology must offer distinct advantages over other implantable physical biosensors in specific biomedical applications. The wireless and passive nature of this technology enables it to be placed in the human body for long periods of time without concerns about battery life. By changing the elasticity of the membrane or flexible substrate, the pressure sensor technology will have the advantage of tunable stress or pressure sensitivity [35]. The use of polymer materials enhances their flexibility and versatility, allowing them to adapt to complex shapes and changing environments. They can be classified into transduction mechanisms that are, for example, piezoresistive, piezocapacitive, piezoelectric, triboelectric, and piezomagnetic [36].

Optoelectronic sensors are based around the measurement of changes in optical properties. These changes come about due to interactions between biometric elements like hemoglobin and the target analyte, like oxygen. Such changes can be in absorption, fluorescence, reflectance, emission, or interference patterns. These sensors are equipped with the capability to accurately detect changes in the way that light is absorbed or reflected. In essence, optical biosensors employ photons to acquire information pertaining to the analyte. However, these photodetectors (PDs) are critical components in medical devices that detect changes in molecule density or structure. These changes are translated into electrical signals, facilitating vital signs monitoring, such as HR, RR, and SpO_2_ [37].

Most of the optical biosensors currently on the market feature a similar design and are generally categorized as flexible wearable sensors. Furthermore, the sample is inserted into the biosensor, and a signal is generated that provides the user with information. In this configuration, biorecognition molecules are usually attached to a surface, and the sample to be analyzed is passed over the surface, causing the recognition molecule to bind to its target. Additional reagents may be included to bind to the captured target, thus increasing the specificity and sensitivity of the measurements. However, it is possible to take measurements on a very small scale, for example, for electronic products such as wristwatches and wearable pulse oximeters. In both cases, light from source enters the body through the skin. The body reveals data from the light reflected onto the detector when the properties of the light change [38]. They are ideal for wearable devices that detect human motion. The primary advantages of these sensors are their wider detection range, simplicity and reduced manufacturing cost, as well as their enhanced durability and adaptability. A comparison of sensor attributes is summarized in Table 1, outlining key differences in their characteristics and performance.

### 3.3. Recent Advancements

Wearable biosensor integration is revolutionizing healthcare, enabling continuous monitoring and personalized insights. Wearable devices facilitate real-time data collection, enabling proactive health management. Given the ongoing challenges in the healthcare sector, these innovative devices are addressing the pressing need for continuous monitoring tools. The wireless platform is the enabler for effective diagnosis and progression tracking. The evolution of biosensor technology has undergone a transformational trend, driven by advances in data acquisition techniques that have pushed portability to new heights.

Presently, researchers are investigating innovative solutions for flexible sensors. Taking that into account, recent studies show progress in two-dimensional (2D) materials for flexible PDs [37], flexible tactile sensors [38], and ferroelectric films [39], with the aim of enhancing the sensitivity, flexibility, and durability of these flexible sensors. Currently, the development of polymers and composite materials is being promoted to prolong the lifespan and reliability of flexible sensors. Furthermore, there is an increased focus on environmentally sustainable materials, including cellulose-based substrates and biodegradable polymers, to address ecological concerns. In a most recent study conducted by Kong et al. [37], they examined the use of printed 2D materials for the fabrication of flexible PDs. The various materials used, and manufacturing processes involved, are outlined alongside a discussion of the applications for which they are suited. However, the performance of printed devices is often found to be in need of improvement. Also, same authors focus on balancing the convenience of printing while maintaining the performance of 2D material flexible PDs. The results indicate that it has been difficult to reliably deposit 2D material nanosheets at the desired resolution (typically between 1 and 100 µm) over large areas through printing. This is due to higher requirements for hardware design and control software within the entire printing system.

In the context of flexible tactile sensors, piezoelectric sensors are a prominent solution due to their self-powered nature, high sensitivity, and rapid response time. These sensors respond to a broad spectrum of stimuli, converting them into electrical signals, enabling the precise detection of objects and touch sensing. Tang et al. [38] provide overviews of the most recent advancements in flexible tactile sensors, focusing on material properties, designs, and the fabrication and application domains. Firstly, flexible piezoelectric materials perform worse than traditional ones. Their performance must be improved through design, modification, optimization, and dynamic regulation. Secondly, designing dense sensor arrays to enhance versatility is growing, but there are crosstalk problems too. Solutions include optimized electrode design, ML algorithms, and identification for improved signal processing accuracy and efficiency. Thirdly, hydrogel is considered for long-term wear and humidity and self-repairing material development, as well as textile clothing. One of the resolving solutions suggests using materials such as silver nanowires, liquid metals, conductive polymers, and carbon-based materials.

Meanwhile, advancements in the field of flexible electronics have resulted in the creation of unique, transformative free-standing ferroelectric films, which have potential applications in sensors, actuators, and energy harvesting. In their study, Pathak et al. explored recent advancements in the fabrication, characterization, and application of ferroelectric films, emphasizing the potential of these films to be incorporated into multifunctional systems for energy harvesting and health signal sensing [39]. These ferroelectric films are difficult to make, and this affects their performance and use in flexible electronics. Thermal stress arises from differences in how much the thin film and its substrate expand when heated or cooled. As the temperature changes during deposition or processing, these differences can lead to significant mechanical stress, which can cause delamination or cracking. Also, intrinsic residual stresses are developed during deposition and not only result from external factors such as temperature changes; they can also affect the electrical properties of ferroelectric materials, leading to reduced stability and increased current leakage.

Numerous studies discuss multimodal data fusion usage for smart healthcare. For instance, Chen et al. conducted a large-scale analysis of the literature in this field based on quantitative approaches. The results show that the number of articles has increased from 1 in 2002 to 220 in 2022, with the majority being published in interdisciplinary journals that combine healthcare and medical research with information technology and AI. In recent years, researchers have shown a high degree of interest in various research topics, in particular cancer prognosis through multi-dimensional data analysis and AI-assisted diagnostics and personalization in healthcare [40].

It is worth mentioning that the authors of [41] explored three of the most common multimodal data fusion techniques, (1) late fusion, (2) early fusion, and (3) the sketch, and compared them in classification tasks, also outlining the various types of data that sensors can collect for a broad spectrum of sensor applications. Possible future directions should focus on establishing a standard for interaction methods and investigating innovative applications for integrated communication modes, as well as focus on the analysis of the interpretation of multimodal approaches.

The abovementioned literature reports indicate valuable additions for recent advancements in wearable devices, though there are still challenges. To tackle these challenges, potential solutions are proposed. It is recommended that model training start with large and diverse datasets to improve the model’s ability to learn and identify patterns. This strategy uses techniques such as information fusion, integrating data from multiple sources for better prediction. Training with substantial and diverse datasets is effective when dealing with data scarcity, and it enhances a model’s ability to identify patterns. Another solution involves the centralized training of a model without raw data sharing, enabling collaboration to enhance a global model.

In this in-depth study [42], the authors explore healthcare sensing technologies and highlight recent innovations such as integrated nanotechnology and the development of wearable devices. In the context of the transportation industry, biosensors were used to monitor the driver and their health by targeting specific health indices. Health symptoms are different for each person and vary depending on the condition. Some common symptoms include irregular heartbeat, high blood pressure, and stroke. Healthcare systems around the world are struggling with the rising cost of medical treatment and services. Remote health monitoring through wearable devices, like smartwatches and fitness trackers, can help reduce the cost of healthcare management and improve results. In short, these devices and the Internet of Things (IoT) technology are a good option for making sure that patients receive medical care on time and that they receive the correct information. A patient’s vital health signs are closely monitored using a range of sensor technologies that are directly relevant to the assessment of the patient’s condition. These vital signs include blood glucose level (BGL), BP, body temperature, SpO_2_, and HR. While the majority of wearable devices focus on a single mode of analysis, the integration of chemical (e.g., lactate, glucose, pH) and physical (e.g., temperature, strain, ECG) sensing modalities in wearable device could provide a more comprehensive assessment of the user’s physiological state [43].

### 3.4. Use in Health Monitoring Context

Today, technological advances have contributed to the field of wearable technology through their ability to miniaturize devices and enhance the precision of biomedical signals. These devices require minimal energy, ensure the security and confidentiality of patient data, are adaptable to various data storage systems, and are cost-effective compared to traditional methods that require patient visits to medical facilities. With that said, the most commercially available wearables for health monitoring are smart wristbands (19%), smartwatches (16%), and wrist sensors (6%) [44].

Biosensor devices provide real-time, continuous data outputs that facilitate visualization of the user’s physiological status and provide the necessary information for informed healthcare decisions. Besides that, the monitoring applications are designed to assess a wide range of health parameters, including BGL, HR, BP, SpO_2_, body temperature, movement, and sleep patterns. In addition, these devices have yet to be fully explored for their potential in sports monitoring and analytics. In the meantime, recent technological developments in the realm of biosensors have introduced novel methods of noninvasive and nonirritating real-time sensing [45].

Birje et al. [46] present a review article about recent advancements in IoT digital health systems. It explores the latest research to establish a classification framework, organizing these technologies according to monitoring strategies, communication protocols, computing methodologies, and energy-efficient mechanisms. The new generations of wearable biosensors are exploring new ways to interface with the epidermis to monitor physiological status [47]. The subsequent evolutionary step in the field of healthcare will be the integration of IoT [48]. For this purpose, IoT has played a significant role in various industries over the past few decades. For this reason, vehicle safety and traffic management systems can benefit from these monitoring devices also serving as a pivotal function in the implementation of biosensing technologies. The exploration of the diverse applications of sensors and the monitoring of real-time vehicle engine status [49] have the potential to highlight the impact on traffic safety when combined with vehicle driver health biosensors. For example, in the modern healthcare environment, where precision and timeliness are paramount, biosensors have emerged as a pivotal element, offering crucial insights that point to the emergence of transformative advancements in preventive diagnostics and proactive personal care. Moreover, with their innovative capabilities, biosensors serve as a crucial asset, facilitating early detection and timely targeted interventions across a wide range of medical domains. With that said, the prospective implications of sensing platforms on monitoring driver state remain fully realized. Broadening the scope of wearable sensors will briefly be introduced in the following section.

### 3.5. Law Regulations

Data privacy, cybersecurity, and the classification of custom-made monitoring devices as medical monitoring tools are things current frameworks should address. Automotive safety standards influence the collection, storage, and use of data for driver safety; meanwhile, the legality of monitoring drivers depends on privacy laws, employee rights, and industry regulations. The regulatory requirements vary by data collected and by region. See Table 2 for key country requirements regarding biosensors.

## 4. Wearable Sensors

Sensors have a wide range of applications, including navigation, automotive technology, and the monitoring of physiological processes. Furthermore, in the domain of healthcare, these sensor devices can be incorporated into wearable micro and nano sensors [50]. For example, a rich composition of wearable technologies based on their function, advanced sensing, and data collection systems are employed for the purposes of managing human health and diagnostics. In the meantime, the rapid detection of driver state is currently of crucial importance for improving road safety and preventing road accidents. As a result, the remote monitoring of vehicle driver’s health can be achieved through the combination of wearable technologies and vehicle telematic systems.

The advent of flexible and wearable electronic devices has significantly enhanced human well-being and propelled the industry to exponential growth in recent times. First and foremost, disposable sensors integrated with electronics have the potential to transform our lives, as these sensors become an integral part of our everyday lives [51]. Furthermore, wearable sensors represent a revolutionary development in the field of medicine, with applications in diagnostics, therapy, and management of diseases. The rich composition of wearable technologies is based on their function, advanced sensing, and data collection systems.

The majority of wearable devices are electronic products designed for use on the body. In the same way, those sensor developments will also result in new roles and responsibilities for patients and healthcare professionals. In addition, regular health check-ups can assist in the identification of any underlying conditions that may impair an individual’s ability to drive safely, thereby ensuring that drivers and passengers are in a secure environment.

**Figure 4:**
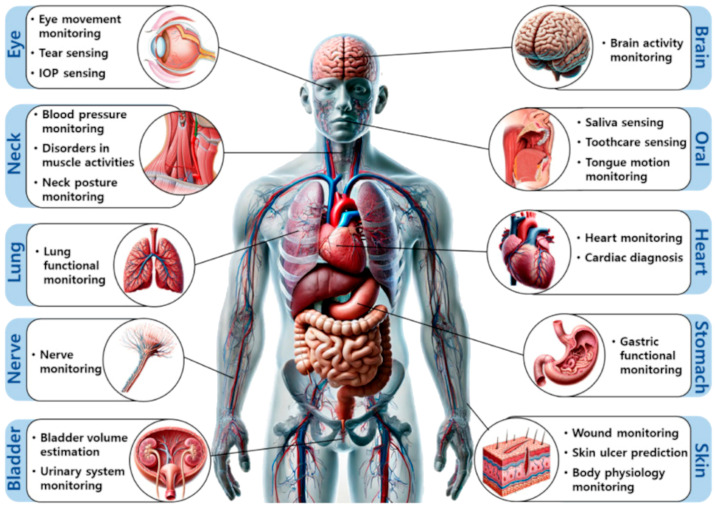
Healthcare sensing applications using wearable sensors. Copyright Elsevier (2025) [52].

Figure 4 classifies the body’s organs according to their respective systems and provides an overview of their primary functions, showing the importance of such an application of sensors. Thereafter, various research in this area has been performed over the last decade.

The authors of [53] presented a framework that elucidates the integration of artificial intelligence (AI) techniques with clinical data obtained through wearable sensors. Meanwhile, the authors of [54] invented a wearable device for the continuous real-time health monitoring of athletes through sweat analysis. As a result, the presented method has the capacity to facilitate the acquisition of knowledge regarding an individual’s health status, including stress level, athletic performance, hydration levels, electrolyte balance, metabolic processes, and even certain medical conditions. Meanwhile, Majumder et al. [55] presented an analysis of diverse wireless technologies and a critical evaluation of their suitability for incorporation in wearable health monitoring systems. Also, the researchers in [56] recently conducted a study on the development of advanced wearable electrochemical biosensors. These biosensors aim to provide noninvasive health monitoring with high sensitivity and flexibility.

The majority of wearable devices fall into four main categories: head-worn, body-worn, lower body, and wrist-worn or handheld. Ironically, these devices, such as smartwatches and wristbands, can be described as primarily fashion items with limited sensory capabilities. For this purpose, biosensor devices are characterized by their robustness, lightweight design, and durability and can convert non-electric physiological activity into electric signals [57]. Meanwhile, Dincer et al. [58] used disposal sensors to describe the integration of compact electronic technologies into diagnostics, food, and environmental monitoring. Ultimately, in terms of future trends and challenges, the development of new disposable device classes using green materials for sustainable, biodegradable, and low-cost production is set to be a key focus.

In essence, the overall goal of the health monitoring system is to provide patients, healthcare professionals, or even healthcare providers with the tools they need to maintain a healthy population. Such services include the capacity to provide feedback messages to systems that provide higher-quality care to a greater number of patients with less risk of burnout while maintaining current control methods and easy access to patient data. Additionally, patients can independently maintain a healthy lifestyle, prevent complications, and minimize personal costs through the use of wearable and mobile devices. In addition, the efficient management of patient and healthcare provider data is facilitated by health monitoring systems.

In another approach, the authors of [59] review the use of wearable and portable sensor technology in epidemiology and clinical medicine and examine how AI is revolutionizing this field. However, as technology develops, managing data appropriately will be key, protecting privacy and preventing the exploitation of sensitive data while allowing researchers to conduct studies in the public’s interest. Moreover, as the shift towards sensor development progress, Heikenfeld et al. [60] state that advertising progress has been made by adapting the available measurement methods. It has involved innovation by miniaturizing and making flexible sensing technologies. However, noninvasive chemical sensors have experienced significant difficulties in application. A major challenge has been the improvement in the mechanical, electrical, and optical sensing modalities. In particular, the specificity of detection has been improved. To understand that, we must consider human skin as an information barrier.

Other key targets, such as vehicle occupants, have also gained substantial attention in wearable biosensors, with a specific focus on fatigue monitoring, as this is determined by heart rate variability [61]. As a result, one of the main conclusions of this study was that an increased heart rate may be triggered by elevated traffic levels. Furthermore, the adoption of an aggressive driving style has been observed when monitoring human motion as this can elevate the level of adrenaline in the body, which causes the driver’s heart rate to increase [62].

### 4.1. Common Types and Key Parameters

In the medical and healthcare fields, sensors are commonly used to measure human physiological indices such as acceleration, heart rate, respiratory rate, temperature, humidity, and SpO_2_. Hence, by measuring these parameters, specific recommendations can be made to improve the general health of the human body. Wearable sensors can be classified depending on whether they measure physical or chemical quantities. For instance, chemical measurements rely on electrochemical, optical, and microelectromechanical angles. Therefore, wearable human movement monitoring devices have good development prospects with the development of IoT and the emergence of various flexible sensors.

To sum up, the factors mentioned below ensure the accurate detection of physiological signals, the reliability of reading under various conditions, and overall efficiency in real-time monitoring and diagnostics. Accordingly, key parameters for electromechanical sensors in medical applications include sensitivity, stretchability, range of detection, response time, linearity, and power consumption.

The first of them is sensitivity, also known as the gauge factor (GF), which is key to developing motion sensing applications, such as pulse monitoring and respiration monitoring. However, strain gauges for tracking finger, arm, and leg joints need to be highly flexible and stretchable. Sensitivity is a quantitative metric that assesses the precision and effectiveness of a sensor; it is expressed as the ratio of the rate of change in the output signal to the applied tension or compression force [63].

Next, stretchability is a prominent factor in wearable sensors. This factor is defined by the elastic modulus of the sensor in the tensile test. The relationship between the elastic modulus and other pertinent variables is expressed by the following equation: E = dσ/dε, wherein dσ is the applied stress and dε is the corresponding strain. Afterwards, the range of detection is the interval between the minimum and maximum levels of stress that the sensor is capable of perceiving.

Also, response time is defined as the duration required for a sensor to generate a stable and measurable output signal in response to an external trigger. It is evident that sensors with reduced response times are more suitable for applications such as the real-time monitoring of vital health indicators. Linearity, defined as the extent of deviation of the output signal from a straight regression line, is another key metric. Linearity is a measure of signal stability over an application range.

Finally, energy usage must be considered when designing a sensor, given its direct relation to the operating voltage. A sensor that operates with minimal energy usage is an optimal choice for integration into mobile devices and those that are difficult to access.

### 4.2. Wristbands

Recently, Campos-Ferreira et al. proposed a human–vehicle interaction monitoring system to supervise the heart status of the driver by using a biometric wristband [64]. In addition, the scientists in [65] made a wearable device in the form of a wristwatch to monitor health status. With that said, the breath analyzer is capable of detecting the biomarkers associated with metabolic processes, where the sweat analyzer can monitor hydration and other health-related indices. Similarly, the identifiable infrared (IR) sensor is capable of measuring temperature or blood flow; meanwhile, rendering data for future analysis might be better, and this can crucially affect these types of devices and their suitable applications for future advancements such as health monitoring, fitness tracking, and even medical diagnostics.

### 4.3. Smartwatches

Smartwatches have quickly grown from fashion items to essential devices for everyday use, with a wide range of functions and applications in the context of health research. Hence, in the transportation sector, smartwatches provide a cost-effective and dynamic way to monitor the driver’s condition and driving behavior.

In the context of classified applications, activity monitoring, chronic disease self-management, home or institutional care, and health education were mainly presented by [66]. With that said, a recently published review outlined the current state of research and development in smartphone-based health technology. The smartwatch Garmin Fenix 5X was selected for its capacity to monitor the heart rate of truck drivers during the working day in timber transportation [67]. Meanwhile, Barka and Politis [68] discussed options of smartwatch use for smartwatch usage data related to driving. Integrating these features with driving analytics could help to understand driving patterns, encouraging more responsible behavior, and ultimately contributing to road safety.

In a most recent study by Carrier et al., they evaluated the accuracy of maximal oxygen consumption (VO_2_) estimates and SpO_2_ measurements taken by the Garmin Fenix 6. Accordingly, the reading was compared with those from a Roscoe Medical Fingertip medical-grade pulse oximeter. The results of this study demonstrate the Garmin Fenix 6’s capability to provide accurate VO_2_ maximal estimates, but above all, they mark huge limitations in accurately measuring SpO_2_, which may restrict its use in specific applications such as monitoring acclimatization and managing pulmonary diseases [69].

### 4.4. Ring-Type Sensors

The integration of an electronic interface, energy source, and wireless data transmission capabilities into compact and lightweight noninvasive platforms necessitates a high degree of integration and miniaturization. As a result, Pacchierotti et al. [70] proposed a wearable device, called “hRing”, which consists of two servo motors that enable the motion of a belt in immediate contact with the user’s finger skin. Also, Poorzargar et al. examined 22 studies of pulse oximeter accuracy under low-perfusion conditions. The findings indicated that the greatest part of oximeters is accurate for patients with poor perfusion, particularly modern oximeters [71].

Meanwhile, a comprehensive list of user awareness of the pulse oximeter in a healthcare context can be found in the reviews of [72,73,74], while Ganesh et al. developed a cost-effective solution for monitoring cardiovascular activity in a portable format [75]. With that said, Mohini Baburao et al. [76] described the application of a ZigBee module, which facilitates the mobility of the monitored patient. Also, a study conducted by Li and Warren [77] described the development of a portable, low-cost pulse oximeter that provides accurate data without the need to use filters. The sensor presented the capacity to gather data of high integrity at various locations on the body, including the fingertips, wrists, earlobes, palms, and even temples.

Meanwhile, Siddiqui et al.’s approach [78] was to provide a convenient, accurate, and distraction-free way to detect driver fatigue in a natural environment. Data consisting of Revolutions Per Minute (RPM), age, and labels were combined into one dataset. A variety of ML models have been trained on the one dataset, of which the Support Vector Machine (SVM) showed the best precision at 87%. Moreover, Volpes et al. [79] developed a device designed to be worn on a finger, with the capability of capturing bio signals directly on the fingertips. This product offers the significant advantage of being both comfortable and easy for users to apply. Meanwhile, the authors of [80] developed a brand new device, and the method involved using inertial data to identify different types of tremors. This is the first study to demonstrate that different types of electromyographic tremors have their counterparts in terms of rhythmic movement.

Specifically, in [81], the authors employed a variety of ML models to train an accelerometer. The following investigation concentrates on the gyroscope sensors of a custom-built smart ring and a smartwatch. Nevertheless, Li et al. developed a model that uses data from a physiological measurement sensor to discriminate between safe and dangerous driving conditions and predicting the probability of transition. It achieves an accurate rate of 90.67% and can be used for the development of proactive strategies to avoid accidents [82]. With that said, Kuczynski et al. [83] designed a device that can measure low-amplitude constant magnetic fields. Within this context, two heuristic approaches are used to estimate the internal properties and perform the regression task. The experiments show that the hysteresis loop has a minimal mean square error, indicating its better suitability for the heuristic modeling of real devices.

Also, Valenti et al. designed and implemented a novel, wearable, multi-sensor, annular probe for the synchronous and real-time acquisition of photoplethysmography (PPG) and galvanic skin response (GSR) signals. It allows for physiological indexes like heart rate, SpO_2_, and their dynamic changes with time. This device makes it easy to detect various physiological states, including rest and exercise [84].

Another example is where Joo et al. demonstrated a simulation model on a finger structure. As a result, calculation was performed of photon intensity through tissue, including the determination of optimal angular separation between a PD and LED detecting SpO_2_. Two different configurations have been numerically and experimentally validated to offer high sensitivity and low energy consumption. Therefore, the accuracy of the models was compared to determine whether smart rings or smartwatches are better suited for gesture detection tasks. Consequently, the system achieves up to 98.8% accuracy using different ML models. All the acquired real-time data were able to process and to predict 12 different gesture classes wirelessly between a smartphone, smart ring, and smartwatch [85].

Over the last decade, an increasing number of customized pulse oximeters have been created. Additional information on oximeter use by various authors can be found in the following sections and is summarized in Table 3.

## 5. Remote Devices and Sensors

With remote systems, the driver does not need to wear any accessories such as a cap, wristband, or pair of glasses. It also ensures continuous health monitoring without reliance on the chance of forgetting to wear the device. To implement remote systems, advanced cameras and sensors must be installed in the vehicle cabin and located towards the right angle to provide a full view of the driver’s face and upper body. Remote devices are more usable, even on long journeys or when there are several passengers in the vehicle cabin. Compared to physiology and face sensors, wearable sensors can fit in any vehicle without having to modify them to fit different drivers [86]. Remote detection is indeed beneficial for commercial vehicles as it employs computer vision and AI to monitor driver fatigue, providing a proactive solution for improving road safety, thereby helping to reduce the risk of accidents caused by sudden driver health issues. The non-intrusive design of unwearable technology ensures a smooth and unobtrusive detection method that preserves the real driving experience while improving traffic safety [87].

Meanwhile, camera-based technology also has been exploited to identify the driver’s state. This approach can be classified based on the indices that require monitoring, such as facial expressions and head movements. Authors from Belgium [88] used eye closures and employed commercially available algorithms for extracting an eye image. This includes facial region detection, facial landmark localization, eye location, and eye capture using advanced image warping techniques. Moreover, Killian et al. [89] reported that the two ECG devices demonstrated greater reliability in comparison to the photoplethysmography devices. Furthermore, seven measures of heart rate variability were collected simultaneously from four different devices during five different behaviors.

To deal with facial expressions, Teyeb et al. [90] developed a multimodal system to detect instances of driver distraction using two visual parameters, namely eye closure duration and head movement angle. This method has been applied to the identification and tracking of the head and eyes, which are deemed to be critical locations of attention. The system is made up of two sub-systems: a head posture sub-system and a sub-system for detecting eye blinks. In another study by Guo et al. [91], two main challenges were addressed for real-time processing and resistance to fluctuations in the vehicle driver’s face. This includes temporal tracking of characteristics over time and a spatial component that extracts facial elements, such as lips and eyes, from a single image. In a study by Deng et al. [92], they addressed a method for drivers wearing glasses in lighting conditions or scenarios.

In a recent study, Essel et al. use technology that detects driver drowsiness in real time by detecting facial features. This study uses an improved algorithm to detect drowsiness when driving long distances. Also, it proposes two methods to assess driver drowsiness. Firstly, static thresholding achieved 89.4% accuracy and 96.5% sensitivity using 1000 images. Meanwhile, adaptive frame thresholding was tested using four 30 min videos from a public dataset, achieving 98.2% accuracy and 64.3% sensitivity using 500 images [93].

In consideration of the driver state, scientists from Jordan [94] have introduced an advanced integrated system based on the Raspberry Pi, which is affecting a revolution in the manner of ECG signal surveillance in clinical application areas. Physiological data (e.g., heart rate, blood pressure, glucose levels) have traditionally been the primary focus of healthcare IoT. But the increasing emphasis on movement and behavior tracking introduces new dimensions of privacy considerations. Remote sensors mostly focus on movement and behavior patterns rather than physiological data.

### 5.1. Smartphones

Smartphone biosensing has two main advantages. It can capture photos of around 1–5 MB each, or 100–600 MB of video per minute, depending on the data format and quality configuration. It also has a clock rate of over 1 GHz. The CPU can be set to a frequency and store (8–512 GB) or transfer (up to 100 MB/s) for 4G mobile networks [95].

Smartphones are increasingly being used to monitor health, using embedded sensors and various applications to track vital indices, such as physical activity, sleeping patterns, and mental health. With functions such as heart rate monitoring, GPS training tracking, and syncing with portable health devices, users can take a proactive approach to managing their health. Sarmadi et al. [96] presented a review article of the latest smartphone sensor practices for health monitoring structures, also covering applications for Android and iOS. It provides a thorough understanding of structural health monitoring (SHM) studies on smartphone sensor technology. Meanwhile, [97,98] reported a review of the literature on smartphone sensing, focusing on the outlines—the types of health outcomes, the types of data, and the challenges faced. Sheikh et al. [99] provide a comprehensive overview of smartphone-based sensing, which can have a positive impact on the management of patient health status. Being aware of condition domains can prevent worsening and further negative effects. Yuan et al. [100] developed a real-time fetal ECG monitoring system based on Android smartphones. The results show that when using the FastICA algorithm, a fast iterative optimization algorithm for processing high-dimensional data, a fetal ECG can be extracted, and the sample entropy correctly detects the signal channel.

Various studies have explored health monitoring systems, focusing on different monitoring types, associated indices, and potential limitations. A comprehensive summary of these sample articles is presented in Table 4, highlighting key aspects of each study and identifying challenges in their implementation.

Overall, implementing an automatic sensor activation feature upon waking could significantly enhance the functionality and usability of smartwatches, providing users with a more integrated health monitoring experience. One of the ways to improve this could be to make smartwatches more like sensors. This would mean that the sensors would start automatically when the person is woken up. This will get rid of the need to press the button, making it a one-handed experience [101].

### 5.2. Wireless Communication in Remote Patient Monitoring

Wireless communications that enhance the provision of health services have been the subject of numerous studies. Zigbee, Wi-Fi, Bluetooth, Bluetooth Low Energy (BLE), LORA, ANT, and UWB technologies are commonly employed as short-range communication technologies in remote patient monitoring systems. Nevertheless, it is imperative to address issues such as clinical validation to guarantee the accuracy and reliability of the data collected by these devices. Furthermore, it is essential that appropriate training is provided to ensure that both patients and their caregivers are adequately instructed regarding the utilization of these devices and the interpretation of the results [101].

A few years later, the collective findings of the review carried out by Amini Gougeh and Zilic emphasize the significance of integrating multiple sensory modalities. The integration of heart rate monitors with motion-detecting sensors can enhance the detection of activity, which, at some point, can lead to improved health outcomes [102].

### 5.3. Next-Generation Smart Devices

Wearable sensors are considered a class of next-generation sensors, with applications in both diagnostics and regular monitoring. Wearable sensors come in many types, from heart rate and blood sugar to movement sensors. Accelerometers, which detect movement, are useful for monitoring everyday activities, especially for the elderly. Wearable sensors also help patients to recover after surgery. Using electrochemical biosensors enables smartphones to connect wirelessly to devices using electrochemical methods. Wireless components are portable, simple to use, and more convenient than wired components. This emphasizes the need for innovative designs and manufacturing techniques for the future [103].

## 6. Challenges and Limitations for Future Applications

Biosensor technology has made huge strides in healthcare, but there are still big challenges to overcome if we want to see it grow even more. It is very important that we tackle these challenges if we want to make the most of biosensors and improve traffic safety and healthcare. With that said, Kurz et al. [104] compare smart rings and smartwatches that use motion sensors for gesture recognition. The move towards smart rings has resulted in a highly mobile and wirelessly connected set of devices. As can be seen in Table 3, there are already many customized ring sensors available, demonstrating their innovative designs and diverse applications.

With all these advances in biosensors, wearing health monitors as remote healthcare tools will have a big effect on road safety by letting us check driver’s health in real time. But there are still some problems stopping these devices from being used by healthcare systems around the globe. Key circumstances include checking that the devices work, that people are accepting the devices, and making them in a way that is cost-effective. Today, most healthcare organizations cannot protect patient information from unauthorized access. While centralized cryptographic solutions have been introduced to secure data, they have not fully resolved the problems. Mahajan et al. introduce a potential solution, blockchain technology, where they present the model of cloud-based electronic health records (HERs) and the applicability and benefits of blockchain [105].

The current discussion of future perspectives is overly generic. To enhance actionable insights, maybe it is better to consider proposing concrete research pathways such as developing multimodal fusion algorithms integrating ECG, PPG, and gaze tracking, and implementing blockchain frameworks for encrypted health data transmission

For example, recently, the authors in [106] proposed an explainable multimodal data fusion approach, combining ECG signals with selected blood test results, to improve heart failure detection. This approach is particularly prevalent in domains such as medical diagnoses, where data from various sources like real-time sensors, medical images, and electronic health records are exploited to predict diseases accurately. In the driver monitoring sector, robust in-vehicle heartbeat detection was developed by Warnecke et al. The results show the potential to use over half of drive time for continuous monitoring with the ECG and PPG sensor and a low variance between the different driving scenarios [107].

In the transportation industry, especially the field of traffic safety, several technical difficulties remain in aspects related to a lack of well-established remote networks, as well as the selectivity, sensitivity, power supply, and cost of a monitoring device itself. All the devices mentioned, except for the remote sensors, are devices whose power consumption is a key performance indicator. However, an analysis by Canali et al. sought to understand the role of wearables in relation to other biomedical technologies and data sources, as well as how to approach their adoption and regulation. It specifies the functions of wearable technology in biomedical research and clinical care and its epistemic contribution to the development and application of biomedical knowledge [108].

### Comparative Analysis

In this concluding subsection, we summarize the advantages and limitations of the wearable and remote sensors discussed earlier. Comparison of those sensor capabilities are shown in Table 5.

Taking into account real-world driving data for heart rate monitoring, several studies indicate the mean error rates for validation. For instance, Vila et al. [109] examined the real-time quality index to control data loss in real-world cardiac monitoring devices. The results indicate high accuracy for the estimate of mean HR (mean error: 3.2%), poor accuracy for short-term HR characteristics (e.g., mean error: 64% for HF power), and low accuracy for long-term HR characteristics (e.g., mean error: 25% for LF power). These errors could be reduced by using the quality index to identify windows with little or no data loss.

In another example, the authors [110] used three wrist-worn consumer products designed for heart rate measurement. The results show that the mean absolute percentage error for heart rate varied from 2.17% to 8.06% for the Fitbit Surge, 1.01% to 7.49% for the TomTom Cardio, and 1.31% to 7.37% for the Microsoft Band. In the same year, Meseguer et al. conducted an analysis to ascertain the correlation between heart rate and driving style. The experimental results demonstrated that there was a significant difference in heart rate between quiet and aggressive behavior, with the latter exhibiting a range of 2.5% to 3% more beats per minute. The same author also highlights a notable correlation between heart rate and driving style, particularly when comparing quiet and aggressive behaviors. The results indicate that individuals exhibiting aggressive driving behavior experience a 2.5% to 3% higher heart rate compared to those with a quiet driving style. This suggests that aggressive driving may induce a greater physiological stress response, as reflected in the elevated heart rate. Such findings could have implications for understanding the impact of driving behavior on stress levels and overall cardiovascular health, particularly in high-pressure or high-risk driving scenarios. Further research might dim the distinction between whether these differences persist across different demographics or driving conditions [111].

The research in [112] summarizes achievements and recent progress made in wearable health monitoring systems. It also presents strategies for material selection, system integration, and signal monitoring. The main features of wearable health monitoring systems are conformability, safety, and stability. Furthermore, artificial intelligence (AI) is a highly effective means for wearable systems, which has been used to analyze sensing data. The reliability of the learned model is contingent upon the database containing effective, sufficient, and authentic training samples, given the direct impact of the algorithm on sample capacity. Consequently, the collection of sample data is an essential prerequisite for establishing a satisfactory database. Moreover, it is imperative to refrain from excessive artificial data correction when employing artificial intelligence for data analysis. The seamless integration of biosensor-generated data holds immerse promise for advancing personalized medicine and data-driven healthcare decision-making. Integration is challenging. For example, connectivity issues and different data formats affect data exchange, while privacy concerns pose a significant risk to human information confidentiality [108]. Robust data standards, encrypted protocols, and compliance with strict privacy regulations are necessary to protect sensitive human information [113]. The rise in point-of-care technologies could make healthcare more accessible by allowing for rapid and cost-effective diagnoses [114].

This article reviews the key implications of using biosensors for vehicle driver monitoring, covering a discussion of the historical development, design and operational principles, and health monitoring in the future. There has been a significant increase in smartwatch use in the road transport industry in recent years. Researchers should note that devices often lose data due to connectivity problems or different placement on the subject. The integration of innovative technologies in the medical, materials, and electronics sectors will facilitate the transition of smart wearables from a niche market to a ubiquitous feature [115].

Despite recent advances promising the successful implementation of electrochemical wearable biosensors, several challenges remain to be overcome, particularly regarding stability, reliability, device integration, and biocompatibility. The proposed approaches are intelligent but could adapt more accurately to the complex and unpredictable driving situations of the real world. In addition, the complexity of the modeling, the cost of computing, and the data processing requirements are major obstacles to their adoption. The potential of wearable sensors to provide reliable measurements has attracted increasing attention from the scientific community. The advancement of device portability and the data processing capabilities will facilitate the realization of accurate health analysis based in the future. This will be achieved through the utilization of wearable sensors and mobile phones for data interaction and the real-time display of medical information.

At present, the most prevalent wearable devices for health detecting are smart bracelets (19%), smartwatches (16%), and wrist sensors (6%) [44]. The range of sensors and materials available for wearable devices makes them suitable for many research needs. However, materials need to be selected not only to meet technical and metrological requirements but also to make economic sense. With that said, the advent of wearable sensors has undoubtedly facilitated advancements in the manufacturing process of such devices; at the same time, adding heart rate monitors can improve the accuracy of driver fatigue monitoring.

In addition, the effectiveness of wearable sensors needs to be scientifically validated. Products using wearables and biosensors have little evidence to support their claimed benefits, and their practical value continues to be debated [112]. As the wearable sensor industry continues to develop, it will be vital to develop solutions that go beyond functionality to address user privacy and resource efficiency. Prospective technologies will contribute to the advancement of the field and provide useful insights for future researchers by mitigating the shortcomings we have outlined and by reducing the difference between different types of driver monitoring systems.

The collaboration between biosensors and applications can facilitate the progress of precision medicine, generating significant financial returns and improving the quality of life of users. Health and performance technology companies should collaborate with scientists to demonstrate the effectiveness and scientific value of their products, enabling the application of biosensors in everyday life.

## 7. Summary and Outlook

In recent years, the field of wearable electronics has undergone rapid development in monitoring capabilities. The principal criteria for the assessment of wearable sensors are data sensitivity, refresh rate, detection radius, and linearity. The parameter of ductility is of particular significance in the evaluation and popularity of wearable sensors; they do not need to be sampled or processed, making them accessible to all.

Summarizing the overall contributions, ring-type oximeters can rise significantly in the global market in transportation companies and in the traffic safety context overall. With that said, biocompatible materials and edge computing also highlight novel contributions in the sensor field. Transportation companies are leveraging advancements in biocompatible materials and edge computing to enhance their presence in the global market, thereby enhancing traffic safety. To improve the energy efficiency of low-power wireless protocols, crucial action steps must be taken to ensure the widespread adoption of IoT devices. Time scheduling must be optimized, and the impact on energy efficiency, latency, and reliability must be evaluated to ensure a balanced approach that considers the full range of needs of IoT applications. Meanwhile, starting with standardized driver health databases, the development of a guide should be considered, starting with specifying sampling rate range and data formats for different types of human health metrics. To improve the accuracy of sensor data, noisy and missing data should be filtered and not be considered at the data processing stage. Establishing benchmarks would be one of the ways to improve data quality and reliability overall. To easily access already revised and edited data, communication protocols such as HTTP should be used, and developing operating software for third-party applications can also pave the way for interoperability across all platforms. With that said, to represent diverse driver populations and driving conditions, additional attention should include data from various driving environments (e.g., urban, rural, highways) and conditions (e.g., day/night, seasonal variations). Furthermore, to facilitate real-time feedback to the driver, edge computing solutions and in-cabin interfaces for alerting drivers should be considered priorities.

The usage of edge computing channels such as Zigbee, Wi-Fi, and Bluetooth has become pervasive within the domain of healthcare systems, owing to their efficacy in facilitating communication over both short and extended distances. Zigbee has gained a particular favor in scenarios where low-power consumption is paramount; its deployment in sensor networks is a testament to this. In contrast, Wi-Fi stands out by its capacity to deliver high data transfer rates, making it an ideal solution for the processing of substantial datasets. The prevalence of Bluetooth can be used for its aptitude for establishing short-range connections with remarkable ease.

In general, after exploring different types of sensors, their operating principles, and their integration into specific applications in different fields, it is clear that wearable sensors have become a crucial tool in medical diagnosis, environmental monitoring, food safety, and security. Wearable devices themselves and their miniaturization properties show premise to transforming healthcare. Wearable biosensors, in the future, might just dim the distinction between the transportation industry and health monitoring itself. Advances in technology are thus creating advancements in customized medicine, using real-time data to develop monitoring operations. The shift towards preventative healthcare is gaining momentum, as wearable biosensors empower vehicle drivers to take proactive steps in managing their health and, eventually, improving their quality of life.

Despite the colossal progress that has been made so far, noncommercial wearable devices that are mostly employed for monitoring health are smart wristbands and smartwatches. Typically, the integration of regular driver health monitoring can serve to mitigate the likelihood of traffic accidents that may be precipitated by underlying health conditions, such as fatigue, stress-related disorders, or sudden health crises. Interdisciplinary collaboration is key to driving advances in functional materials for medical diagnostics. Joint initiatives between researchers in materials science, bioengineering, computer science, and clinical medicine are vital for developing wearable health monitoring devices that provide real-time information on the health status of vehicle drivers.

Finally, with these challenges overcome, choosing biosensors for vehicle driver monitoring could resolve some of the problems faced in the transportation sector. While sensitivity and data security remain significant concerns in healthcare, the development of strategic solutions such as advanced encryption methods, data management, and artificial intelligence for predictive analytics can enhance patient outcomes. By fostering technological advancement in the transportation industry, biosensors such as heart rate and body temperature systems will play a vital role in monitoring vehicle driver health.

## Figures and Tables

**Table 1:** Comparative summary of sensor attributes.

Attributes	Electrochemical	Pressure	Optical	Ultrasound	Magnetic
User comfort rate	Moderate	Moderate	High	Moderate	High
Portability rate	Moderate	High	Very High	Low	High
Set-up time, minutes	15–60+	1–5	15–45	3–15	1–5
Process time, sec.	1–60+	0.1–1	0.1–1	10–60+	0.1–1
Long-term stability, years	1–5	5–15	5–20	5–10	10–30
Data sensitivity rate	High	High	High	Moderate	Moderate
Signal-to-noise Ratios, dB	50–80	60–100	70–120	60–110	4–80
Anti-interference rate	Moderate	High	High	Moderate	Low
Cost, dollars	10–500	5–300	10–1000+	100–10,000+	5–500+

**Table 2:** Law regulations around the world.

Region	Relevant Regulations	Key Requirements
USA	FMCSA, HIPAA, BIPA (Illinois), CCPA (California)	Obtain driver consent, ensure data security, avoid discrimination.
EU	GDPR, EU Mobility Package	Require explicit consent, justify necessity, protect personal data.
UK	UK GDPR, Health & Safety Act	Same as GDPR, must align with worker protection laws.
Canada	PIPEDA (Federal), Provincial Privacy Laws	Consent required, data encryption mandatory.
Australia	Privacy Act 1988, Workplace Safety Laws	Strict regulations on biometric data collection.
China	PIPL (Personal Information Protection Law)	Government approval may be required for biometric data use.

**Table 3:** A selected sample of custom ring-type sensors mapped onto research domains.

The Goal	Experimental Set-Up	Results	Health Indices	Ref., Year
To develop finger-wearable cutaneous device	Device is composed of a static platform that houses two servomotors, two pulleys, and a belt	20% improvement in performance and a 47% improvement in perceived effectiveness for time and effort completion	Finger tracking	[70] 2016
To enable real-time monitoring of patient health data via an online site	ESP8266 microcontroller OLED module MAX30100 oximeter module	Provided a low-cost device and platform with accurate readings	HR SpO_2_	[75] 2022
To extend healthcare from hospital to portable devices	ZigBee module MSP430FG437 processer LM35 sensor	Created system allows portable real-time continuous patient monitoring	SpO_2_ Body temperature	[76] 2012
To present pulse oximeter for diagnostic algorithms	Microcontroller Printed circuit LED module Signal sampling	Shows potential of using research platforms for the extraction of new physiological parameters	PPG	[77] 2011
To design a device on a finger, allowing all biosignals to be captured directly on the fingertips	STM32L432KC microcontroller Three data process buses MAX30102 waveform sensor MPU6050 digital sensor BLE communication protocol	Perceived changes between rest and exercise conditions, also assessing them to individual physiological status	ECG PPG GSR Motion signal	[79] 2024
To develop a new wearable device and method for differentiating alternating from a synchronous rest tremor pattern using inertial data	ISP-1807 module nRF52840 microcontroller LM6DSL IMU 32 KHZ and 32 MHZ crystal clocks BLE antenna	First ever attempt to characterize muscle behaviors, commonly assessed by electromyographic approaches using inertial data and the combination of such data in ML	Rest tremor	[81] 2023
To develop a prototype from a wearable biomedical device, which is capable of acquiring synchronous signals	GSR-MIKROE2860 and MAX30102 ring-shaped sensor probe SMT32-F446RE a microcontroller-based system	System demonstrated to be efficacious in the monitoring of physiological states and the assessment of emotional arousal and oxygen saturation	PPG GSR HR SpO_2_ GSR	[84] 2023

**Table 4:** Summary of sample articles with health monitoring type, the indices, and limitations.

Sample Size	Type of Sensors	Indices	Applications/Devices/Techniques	The Goal	Limitations	Reference, Year
Three healthy male volunteers	Lactate sensor, ECG sensor	Lactate ECG	Custom-made hybrid device	To set up a more comprehensive fitness monitoring system than physical or electrophysiological sensors alone	Data-related problems, mainly to data loss and sweating	[43] 2016
Seventy-six records	Smartwatch	Driver state assessment Benchmark performance Driver assistance systems Detection of driving events	Five-stage methodological framework	To evaluate the extent to which smartwatches have been incorporated into driving-related research	Collect data on long-term health, such as sleep, resting, and physical activity HR	[68] 2024
Five different biomarkers	Amperometric biosensor	Uric acid Creatinine Glucose Lactate	Biosensor was screen-printed directly onto soft bandage fabric, followed by functionalization of the working electrode	To develop a wireless smart bandage biosensor for uric acid	Operational stability of smart bandage exposed to 400 μM over 8 h	[80] 2015
Twenty-five drivers	Eye-tracking glasses	Electrocardiogram blood pressure The eye movement	The experimental route was 26.6 km and included urban freeways, arterials, collectors, and intersections	To collect driver’s physiological data to assess driving risk during lane changes	Data outside the range of the training dataset cannot be processed by the proposed model	[82] 2019
Fifty drivers	Empatica E4 wristband Polar H10 chest band Netatmo device	Driving stress Temperature Humidity Carbon Dioxide (CO_2_) level	25 min drive using a simulator	To analyze how driver’s mental state elements and CO_2_ concentration inside the vehicle affect driving	It did not consider variables such as personality, gender, education level, or driver history	[87] 2020
Eight healthy volunteers	PPG inertial measurement unit	Stress events	Three different kinds of experimental driving scenarios: urban, interstate, and rural	To predict driver stress level by evaluating the steering wheel’s motion pattern	During the driving test, participants cannot change the position of their hands on the steering wheel	[88] 2018
Fourteen healthy participants	Four heart rate sensor devices	Seven measures of heart rate variability in five behavioral conditions	Simultaneous recording of ECG via 4 pathways	To evaluate ECG data measurements between different devices	The behavior of an injured or diseased central nervous system can be highly variable	[89] 2021
Twelve drivers	Two algorithms: static and adaptive thresholding	Eye closure and mouth aperture ratios	Two algorithms: a static and adaptive frame threshold	To design efficient, real-time drowsiness detection algorithms leveraging behavioral parameters	Future research will explore the integration of blockchain to enhance privacy and secure communications in (IoT) networks	[93] 2024
Pregnant woman	Low-energy portable fetal ECG collector	Fetal ECG Maternal ECG Abdominal mixed signals	Real-time display of fetal ECG waveform and fetal heart rate by implementing the fetal ECG extraction algorithm on the smartphone software	To develop an Android smartphone-based ECG monitoring system	Segment of the fetal ECG signal that was obtained by the algorithm was seriously contaminated by noise	[100] 2019

**Table 5:** Sensor differences in various aspects.

Aspect	Wearable Technology	Remote Technology
User comfort	Advantage when using in sports	Limited by water permeability
Portability	Highly portable; worn directly on the body and designed for continuous wear	Not typically worn; can be portable (e.g., remote controls, smartphones) or fixed in a location (e.g., security cameras)
Long-term stability in real driving scenarios	Sensors may degrade; battery life decreases Limited by hardware and integration with vehicles Must withstand physical wear and tear, requires regular charging and potential repairs	Requires consistent connectivity and updates Dependent on vehicle hardware and software
Data sensitivity	Can be affected by poor skin contact, motion artifacts (sports) and skin tone, Sensitive to ambient conditions (e.g., light, temperature)	Data transmitted over long distances can be affected by signal interference or loss, latency or delays in data transmission, and power failures can disrupt data collection
Signal-to-noise ratios	Dependent on user behavior and sensor placement	Dependent on electromagnetic interference, signal attenuation, environmental factors
Anti-interference capabilities	Shielding, advanced algorithms, improved sensor design, redundant sensors	Error correction, frequency hopping, shielding, redundant systems
Cost	Can range from affordable fitness trackers to high-end smartwatches	Varies widely, remote controls are inexpensive, while biosensors prices came up in recent years
Sensing range	From centimeters up to a few meters (e.g., proximity to the skin or body)	Can cover a broader range depending on the type (from meters to kilometers)
Battery life	From a few days to a week	From weeks to months or even years
Data storage and analysis	Data are stored locally on the device or synced periodically to a mobile device	Data are often stored remotely, in the cloud or on a server
Material and component availability	Flexible materials like conductive fabrics, flexible printed circuit board (PCB), silicone, and soft plastics	Metal enclosures, robust plastics, and non-flexible PCB
Real-time data accuracy	Provides instant feedback to users	Feedback is often not immediate to the user but may be relayed to a central monitoring system or a control center in real time
Data acquisition and processing	Edge processing is common, meaning data are processed on the wearable device or transferred to a mobile app or a nearby device for processing (e.g., using onboard microprocessors)	Usually send raw data to a centralized processing system (cloud, server, or local control center)

## Data Availability

Not applicable.

## Abbreviations

The following abbreviations are used in this manuscript:

HR	Heart rate
IoT	Internet of Things
EEG	Electroencephalogram (Brain Activity)
EOG	Electrooculogram (The Cornea-Positive Potential)
CAN	Controller Area Network
ECG	Electrocardiogram (Electrical Activity of Heart)
PCB	Printed circuit board
ML	Machine learning
VO_2_	Maximal oxygen consumption
IR	Identifiable infrared
RAM	Random-Access Memory
PDA	Personal Digital Assistant
LED	Light-Emitting Diode
CAN	Control Area Network
PD	Photodetector
RPM	Revolutions Per Minute
RR	Respiration rate
CO_2_	Carbon dioxide
SVM	Support Vector Machine
BGL	Blood glucose level
GF	Gauge factor
DC	Direct current
SHM	Structural health monitoring
PPG	Photoplethysmographic
GSR	Galvanic Skin Response
